# Rapid and retrievable recording of big data of time-lapse 3D shadow images of microbial colonies

**DOI:** 10.1038/srep10061

**Published:** 2015-05-15

**Authors:** Hiroyuki Ogawa, Senshi Nasu, Motomu Takeshige, Mikako Saito, Hideaki Matsuoka

**Affiliations:** 1 Microbio Corporation, 6-6-3 Minamiyoshinari, Aoba-ku, Sendai, Miyagi 989-3204, Japan; 2 Sendai National College of Technology, Department of Intelligent and Electronic Systems, 4-16-1 Ayashi-Chuo, Aoba-ku, Sendai, Miyagi 989-3128, Japan; 3 Sendai National College of Technology, Advanced Course of Information Electronic System Engineering, 4-16-1 Ayashi-Chuo, Aoba-ku, Sendai, Miyagi 989-3128, Japan; 4 Tokyo University of Agriculture and Technology, Department of Biotechnology and Life Science, 2-24-16 Naka-cho, Koganei, Tokyo 184-8588, Japan

## Abstract

We formerly developed an automatic colony count system based on the time-lapse shadow image analysis (TSIA). Here this system has been upgraded and applied to practical rapid decision. A microbial sample was spread on/in an agar plate with 90 mm in diameter as homogeneously as possible. We could obtain the results with several strains that most of colonies appeared within a limited time span. Consequently the number of colonies reached a steady level (N_stdy_) and then unchanged until the end of long culture time to give the confirmed value (N_conf_). The equivalence of N_stdy_ and N_conf_ as well as the difference of times for N_stdy_ and N_conf_ determinations were statistically significant at p < 0.001. N_stdy_ meets the requirement of practical routines treating a large number of plates. The difference of N_stdy_ and N_conf_, if any, may be elucidated by means of retrievable big data. Therefore N_conf_ is valid for official documentation.

Cell division potentiality is the evidence of cell viability. Repeated cell divisions generate colonies with a diameter in the visible range of millimeters after long culture. In fact, every reference method, including ISO (International Organization for Standardization) methods and AOAC Official Methods of Analysis^1^ is based on a visual count of colonies. In other words, every method stated as a reference method requires long time to obtain colony count results. For practical use, the colony count had better be determined as rapidly as possible without loss of reliability equivalent to reference methods.

To date various rapid methods have been developed using non-culture or micro-colony culture systems^2^,^3^,^4^,^5^,^6^,^7^,^8^. Staining of viable cells with a specific fluorescent dye, for example, such as fluorescein diacetate derivatives^9^,^10^,^11^ and fluorescent glucose^12^,^13^ can be used to determine the number of viable cells rapidly within half an hour, requiring no time for growth verification. On the other hand, micro-colony count is based on culture on an agar plate and is able to provide count result relatively fast by using a microscope imaging system^14^. Those methods have been introduced as being useful enough for microbial test such as a hygiene self-check. However neither of them has been considered as equivalent as the standard reference method. This is because these rapid methods cannot exclude the possibility of false signals to get into the test process and to cause miscount.

Time-lapse measurement of colony growth might be the best way to distinguish viable cells from dead cells and non-biological particles^15^,^16^. London *et al.* developed an automated system for the rapid colony enumeration, utilizing time-lapse measurement of colony growth^17^. To obtain a clear colony image, however, they had to take special care for in-focus measurements. In their set-up, the colonies were formed on a membrane filter placed on a carefully prepared flat agar medium. Recently Levin-Reisman *et al.* developed a more user-friendly system with downloadable software^17^. Their system is based on the time-lapse measurement of colony diameter of every colony on the entire plate with a standard size (ca. 90 mm). Such a system should be useful for personal uses in research fields. However it might not be applied to practical purposes in which a much larger number of plates containing a wide range of cell density need to be measured simultaneously. Moreover rapid decision of the number of colonies is intensively required under those practical conditions.

To meet these practical requirements, we developed a unique automated system for acquiring the 3D shadow image of every colony in the entire culture plate of 90 mm in diameter by single capture shot^18^. Time-lapse shadow images are captured automatically in a multi-focus mode while the plates under test are being incubated under traditional culture conditions. This time-lapse shadow image analysis (TSIA) enabled the distinction of a single colony generated from a single cell, a doublet colony generated from 2 cells, and also a colony attached to a non-biological particle. Consequently the count loss due to such colony fusion could be avoided.

In this study we have upgraded the system so that big data of every colony in 100 agar plates can be stored and shown on a display by real-time processing. Then we applied the system to the colony measurement of 4 strains and 2 contaminated food samples in order to demonstrate its performance of rapid decision of the number of colonies in a wide range of cell density.

## Results

**Figure 1:**
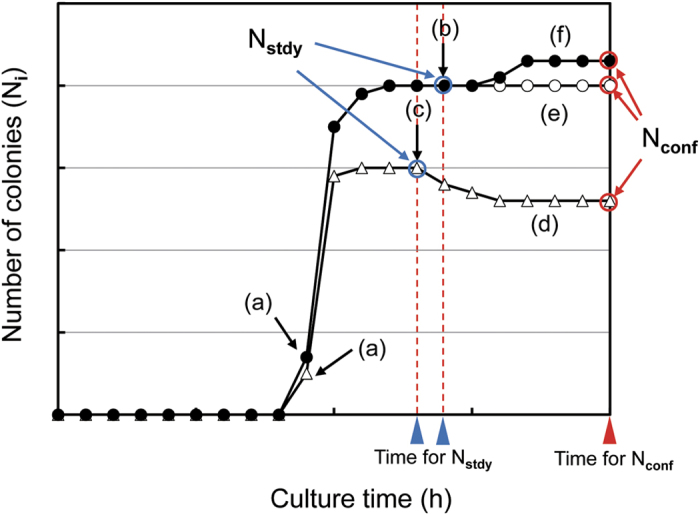
A theoretical model of time courses of the number of colonies, (**a**) initial recognition of colonies, (**b**,**c**) time point of reaching a steady level (N_stdy_) of N_i_, (**d**) occurrence of delayed decrease of N_i_, (**e**) unchanged steady level, (**f**) delayed increase of N_i_. Blue open circle: N_stdy_ values, red open circle: N_conf_ values, blue triangle: times for N_stdy_ determination, red triangle: time for N_conf_.

A theoretical model of the time course of N_i_ is illustrated in Fig. 1, where N_i_ is the number of colonies at image capture time i. In this model, the initial colony is detected at (a). Then N_i_ increases sharply and approaches a steady level at (b) or (c). According to the criteria described below under the paragraph: criteria of reaching a steady level, N_i_ at (b) or (c) is decided to be a steady value, N_stdy_. If N_i_ is unchanged until the end of long culture time (e), N_stdy_ can be an accurate rapid result and regarded as the confirmed value (N_conf_). Otherwise there are occasions of (d) or (f). The decrease of N_i_ will occur because of the fusion of more than 2 colonies into a large colony. On the other hand, the increase of N_i_ will occur when slowly growing colonies appear. In either case, the cause of the difference between N_stdy_ and N_conf_ should be investigated. Stored data are retrievable and useful for its elucidation.

**Figure 2:**
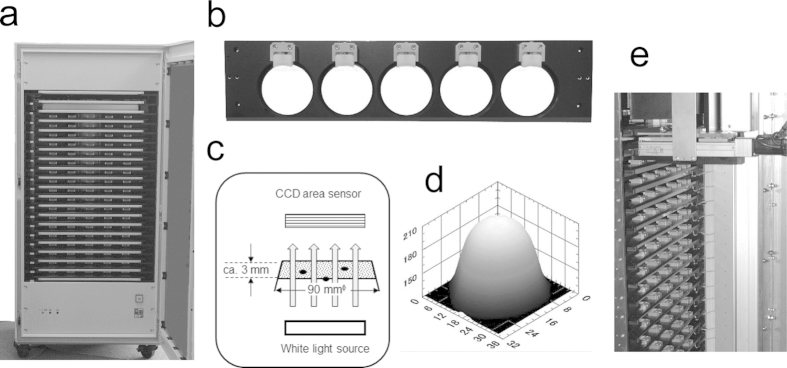
TSIA system. (**a**) Temperature controlled incubator module. (**b**) 5-Plate tray. (**c**) Shadow image capture system. (**d**) A 3D shadow image of a single colony. (**e**) Plate tray convey system installed inside of the incubator module.

We have upgraded a formerly developed system so that it can treat 100 plates simultaneously. Twenty trays were placed in an incubator module (Fig. 2a) and the temperature of each tray was adjusted for growth of the microorganism concerned. The temperature was 35 °C for *E. coli* and colony forming bacteria in general, 33 °C for *B. pumilus*, and 28 °C for *C. albicans* and *A. brasiliensis*. Five plates were set on a tray (Fig. 2b). White light was illuminated to project shadow images of colonies on a CCD area sensor (1536 × 1536 pixels for 100 × 100 mm^2^) (Fig. 2c) and a 3D shadow images of each colony in or on an agar medium was recorded and analyzed (Fig. 2d). TSIA was conducted for every colony image in every plate at prescribed time intervals (usually 30 min or 1 h). Each of 20 trays was conveyed to the camera position by turns with a positional precision of 10 μm (Fig. 2e).

This study focuses the rapid decision of the number of colonies. From this viewpoint, there are 2 points to be considered. One is the decrease of the number of colonies which should occur when more than 2 colonies fuse into a large colony. The other is the delayed appearance of new colonies after most of colonies have appeared. The former point was solved by TSIA as reported previously^18^. The latter point, however, remained unsolved.

**Figure 3:**
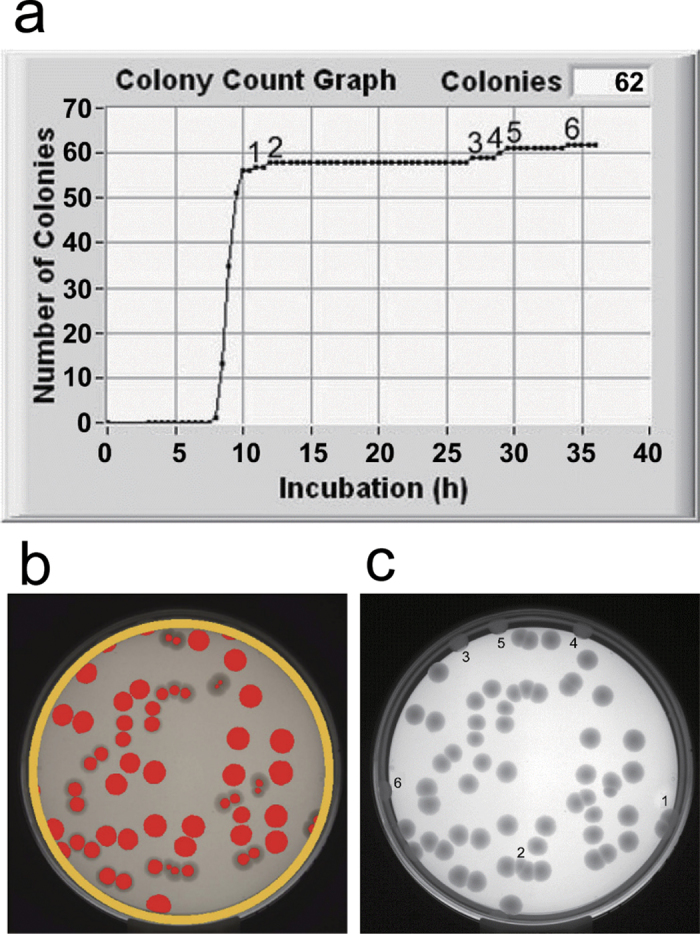
A typical case of delayed appearance of *E. coli* colonies. (**a**) time course of N_i_, (**b**) recognized colonies designated by red markers, (**c**) image data recorded at the end of culture.

A typical case of the delayed appearance of colonies is shown in Fig. 3. Those colonies were located in a narrow rim area and seemed to grow horizontally, suggesting the growth from a cell attached on the side wall of the petri plate. The colony growth from such cells was somehow retarded. This case should become a cause of disagreement between N_stdy_ and N_conf_,. Therefore homogeneous plating is thought to be important. Another point is the influence of colony location on or in the agar plate. In case of aerobic bacteria, the growth in the agar will be slower than that on the surface. In this viewpoint, the spread culture will be better than pour plate culture.

**Figure 4:**
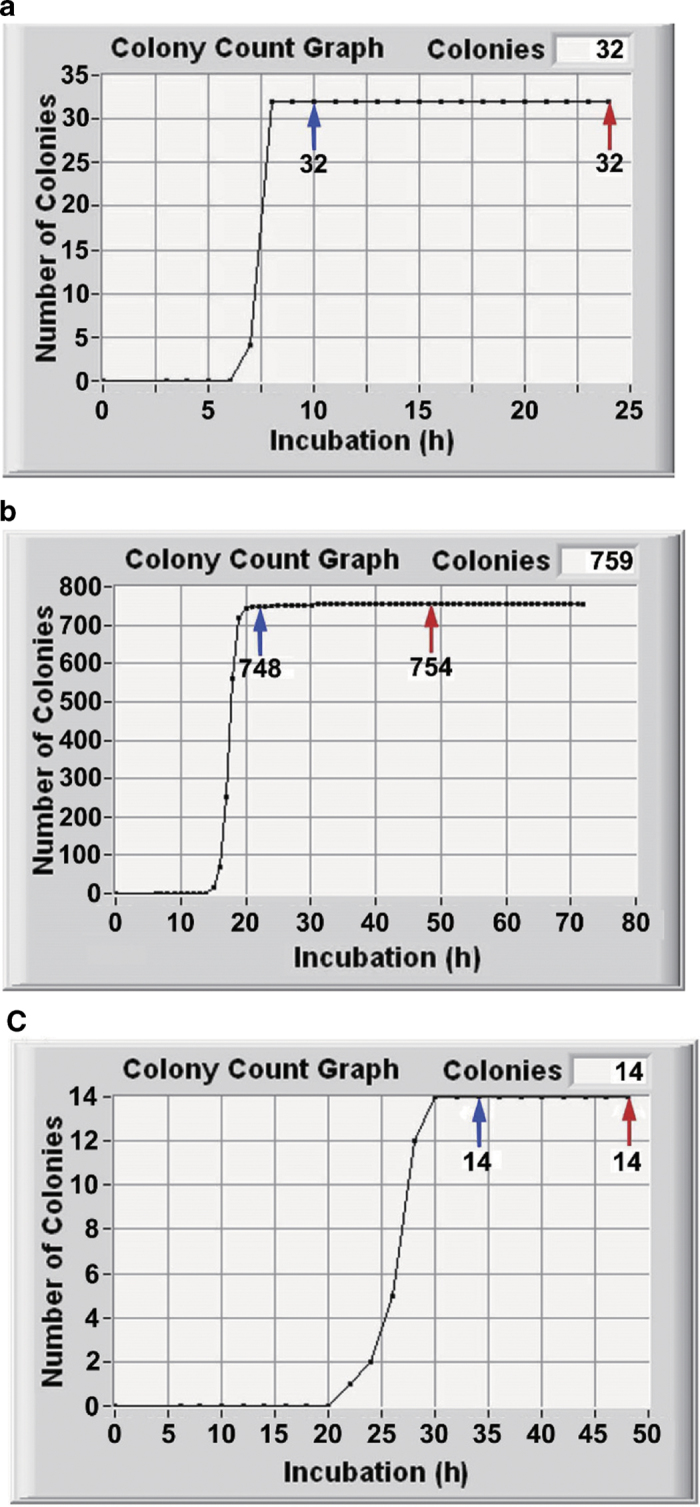
TSIA data of pure culture samples. Time courses of N_i_ of (**a**) *E. coli*, (**b**) *C. albicans,* (**c**) *A. braciliensis*. Blue arrows: N_stdy_, red arrows: N_conf_.

Taking care for homogeneous plating of a cell suspension in/on agar plates, we could obtain successful results with *E. coli*, *C. albicans*, and *A. brasiliensis* (Fig. 4a–c). After reaching a steady level, N_i_ was unchanged until the prescribed long culture time (24 h for *E. coli* and 48 h for *C. albicans* and *A. braciliensis*). Visual count was also conducted and its results were same as those by TSIA for *E. coli* and *A. brasiliensis*. However visual count was too difficult to make a reliable decision for *C. albicans* because of its large number.

**Figure 5:**
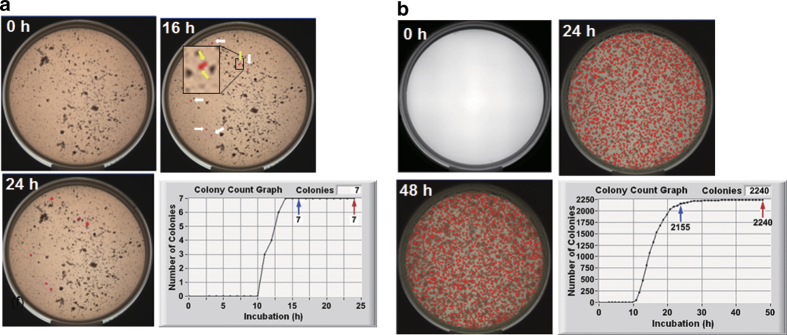
TSIA data of samples containing food matrices. (**a**) *E. coli* in hamburger. Red markers indicated by white and yellow arrows in the panel of 16 h: registered colonies, a yellow arrow indicates a doublet colony and 2 colonies can be recognized in a magnified inset; red markers in the panel of 24 h: registered colonies; blue and red arrows in the time course of N_i_: N_stdy_ and N_conf_, respectively. (**b**) Naturally contaminated bacteria in cut vegetables. Red markers in the panels of 24 h and 48 h: registered colonies; blue and red arrows in the time course of N_i_: N_stdy_ and N_conf_, respectively.

The N_stdy_ and N_conf_ agreements could be obtained successfully also for samples containing a large number of noises such as food fragments (Fig. 5a,b). The images of an agar plate of hamburger captured at 0, 16, and 24 h are displayed in Fig. 5a. Though a number of small particles existed in the plate at 0 h, 7 colonies were counted correctly at 16 h and this value (N_stdy_ = 7) did not change until the end of 24 h culture (N_conf_ = 7). Another food matrix was a sample of cut vegetables (Fig. 5b). There appeared a large number of colonies at 24 h and the number of colonies was 2155 (N_stdy_). This number increased and finally became 2240 (N_conf_) at the end of 48 h culture. In this case the difference was (2240-2155)/2240 = 3.8%.

**Figure 6:**
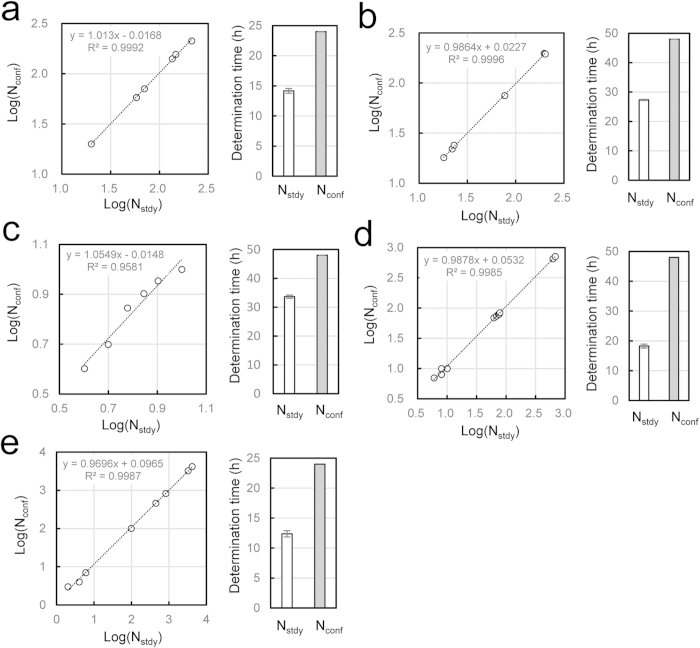
Linear correlation between N_stdy_ and N_conf_ and their determination times. (**a**) *E. coli*, (**b**) *C. albicans*, (**c**) *A. braciliensis*, (**d**) *B. pulmilus*, (**e**) *E. coli* in hamburger. Left panels: Linear relationship between N_stdy_ and N_conf_, determined by least mean square method. Number of samples: 6 (*E. coli*, *C. albicans*, *A. braciliensis*), 11 *(B.pulmilus*). 8 (*E. coli* in hamburger). Right panels: difference of times for N_stdy_ and N_conf_ determination. Gray bars indicate fixed values, 24 h or 48 h. Error bars: mean±SEM.

The equivalence between N_stdy_ and N_conf_ was investigated statistically for 4 pure strains (3 strains shown in Fig. 4a–c and *B. pumilus*) and 1 contaminated food sample (*E. coli* in hamburger). In the case of pure strain of *E. coli*, the linear least square approximation makes a formula: Log[N_conf_] = 1.0055*Log[N_stdy_] − 0.0073 (Fig. 6a). The correlation coefficient was estimated as r = 0.9998 (R^2^ = 0.9997) and degree of freedom was 5. Therefore the equivalence of both results was statistically significant at p < 0.001. On the other hand, the difference of times for N_stdy_ and N_conf_ determinations were also statistically significant at p < 0.001 by t-test. In the same manner, the N_stdy_, N_conf_ equivalence as well as the time difference between N_stdy_, N_conf_ determinations were statistically significant at p < 0.001 for the other cases (Fig. 6b–e). Such a good correlation was obtained in a wide range from 4 cells (log4 = 0.602) (Fig. 6c) to 4140 cells (log4140 = 3.617) (Fig. 6e).

## Discussion

Conventional methods for microbial colony measurement are based on the combination of an appropriate agar medium and culture conditions such as temperature and culture time. It does not depend on whether it is a reference method or a non-validated method. The present system is for automatic processing of those conventional methods and not for proposing a new method based on a new principle. Therefore we have presented experimental data for verification^19^ of the performance of the system rather than for validation^19^ of a new method.

For automatic processing, it is necessary to define a threshold size of a colony. In conventional methods there is no formal criterion of colony size except a remark in ISO 4832^20^. In fact, the colony count results determined by different personnel are often different^21^,^22^. Operators’ skill dependency is one of major causes of uncertainty in colony count method. In the present system, the threshold size was set as 65 μm, because such a micro-scale size was thought to be applicable to wide spectrum of strains in common, though a comparison with reference strains might be necessary. This threshold size was effective for every strain tested here and operators’ skill dependent uncertainty in colony count could be eliminated by automatic processing.

A more serious problem causing measurement uncertainty is complex patterns of colonies after long culture. As long as only one image captured at the end of culture is concerned, it is impossible to determine accurate number of colonies. To solve this problem, retrievable data of time-lapse image should be effective. Thus we developed a system for storing a large number of image data of every colony from its initial appearance throughout until the end of long culture time. Here we have upgraded the system so that entire data of every colony of 100 plates could be processed automatically at practically reasonable speed.

Thus accumulated data may be called big data in comparison with the quantity of data treated by other systems so far developed for microbial colony measurement. As demonstrated above, big data of time-lapse 3D shadow images were really effective for the accurate determination of N_i_ at any time. Operators can easily make a decision of reaching a steady state by watching the real-time display of N_i_ time course on a display as well as by automatic processing according to the criteria defined in the online method section. Thus determined N_stdy_ may be used as a rapid prediction with sufficient accuracy for routine tests in self-check management. Successive recording of the data might not be necessary only for making a rapid decision. However they will be found to be important in case official documentation happens to be required. Then N_conf_ will be used because N_conf_ can be understood to be the result of a reference method if the medium and the culture conditions were equivalent to those of the reference method.

In conclusion the big data based microbial measurement is believed to be an innovative and proactive methodology. In spite of big data processing, a practically reasonable system has been accomplished by unique design of software as well as of hardware.

## Online Methods

### Culture of microorganisms

*Escherichia coli* (NBRC 3972), *Candida albicans* (NBRC 1594), and *Aspergillus brasiliensis* (NBRC 9455) were obtained from the National Institute of Technology and Evaluation - Biological Resource Center (NBRC) (Kisarazu, Chiba, Japan). *Bucillus pumilus* (ATCC 27142) were obtained from ATCC. Each strain was suspended in a medium containing glycerol and frozen at a temperature lower than −35 °C for storage.

Before use, each strain was taken out from a freezer and placed at room temperature. After thawing, the strain suspension was transferred to a tube containing peptone water (Becton Dickinson and Company) for initial culture. *E. coli* was streaked on desoxycholate agar plates. *C. albicans* and *A. brasiliensis* were streaked on potato dextrose agar (PDA) plates. *B. pumilus was* streaked on soybean-casein digest agar with lecithin and polysorbate 80 (SCDLP agar) plates. Every medium was purchased from Eiken Chemical Co. Ltd., Tokyo. The petri plate size was 90 mm in diameter.

### Preparation of contaminated food samples

Hamburger samples contaminated with *E. coli* were prepared as follows. An aliquot of a pure culture of *E. coli* was added to the meat separated from hamburger and stomached for 1 min. A 1 ml aliquot of the resulting suspension was poured into a petri plate and then 20 ml desoxycholate agar medium was added to the plate followed by fully mixing. Cut vegetables were purchased from a market and stomached for 1 min in a phosphate buffered saline (PBS). The resulting suspension was diluted stepwise with PBS. A 1 ml aliquot of the second highest diluent was poured into a petri plate and then 20 ml SCDLP agar medium was added to the plate followed by fully mixing.

### Time-lapse shadow image analysis (TSIA)

TSIA program was developed previously for the acquisition of 3D shadow images of microbial colonies^18^. The shadow image of every colony in a 3-mm thick agar layer could be sharply focused on CCD by means of a multi-focus system^23^. Viable colonies can be distinguished from dead colonies and non-biological particles by time-lapse analysis. The spatial analytical precision is 1 pixel size, i.e. 65 × 65 μm^2^and this size was defined as the threshold size of a colony. More than 2000 colonies per plate could be counted accurately. Using an air-tight bag for a plate, anaerobic bacteria can also be measured. In fact the growth of *Clostridium perfringens* and *Clostridium sporogenes* could be measured (unpublished data). Practically there is no data limit and therefore slowly growing organisms can be measured for sufficiently long culture time.

### Criteria of reaching a steady level

The image of colonies and the number of colonies were recorded at each image capture time and designated as N_i_ (i = 0, 1, 2,···). When N_i_ satisfied the following criteria (1) or (2), N_i+2_ was assumed to reach a steady level and this level was designated as N_stdy_:

1. N_i_ > 100, |N_i+1_-N_i_| ≤ 0.01 × N_i_ and |N_i+2_-N_i+1_| ≤ 0.01 × N_i+1_;
2. N_i_ ≤ 100, |N_i+1_-N_i_| ≤ 1 and |N_i+2_-N_i+1_| ≤ 1.

On the other hand, N_i_ confirmed at the end of long culture time was designated as N_conf_.

## Additional Information

**How to cite this article**: Ogawa, H. *et al*. Rapid and retrievable recording of big data of time-lapse 3D shadow images of microbial colonies. *Sci. Rep.* **5**, 10061; doi: 10.1038/srep10061 (2015).

## References

[b1] AOAC International Microbiological Method Committee. In, Official Methods of Analysis of AOAC International 19th Edition. (ed Latimer G.W. Jr ). Ch.17, 1–279 AOAC International 2012).

[b2] Garry E., Ouattara G., Williams P. & Pesta M. Enumerating chromogenic agar plates using the color QCount automated colony counter. J. Rapid Meth. Auto. Microbiol. 17, 46–54 (2009).

[b3] Clarke M. L. *et al.* Low-cost, high throughput, automated counting of bacterial colonies. Cytometry A. 77, 790–797 (2010). 20140968 10.1002/cyto.a.20864 PMC2909336

[b4] DeJong I. G., Beiharz K., Kuipers O. P. & Veening J.-W. Live cell imaging of *Bacillus subtilis* and *Streptococcus pneumonia* using automated time-lapse microscopy. J. Vis. Exp. 53, 1–6 (2011). 10.3791/3145 PMC3197447 21841760

[b5] Julou T., Desprat N., Bensimon D. & Croquette V. Monitoring microbial population dynamics at low densities. Rev. Sci. Instrum. 83 (2012) 10.1063/1.4729796. 22852704

[b6] Mertens L., Van Derlinden E. & Van Impe J. F. A novel method for high-throughput data collection in predictive microbiology: optical density monitoring of colony growth as a function of time. Food Microbiol. 32, 196–201 (2012). 22850393 10.1016/j.fm.2012.04.001

[b7] Salam F., Uludag Y. & Tothill I. E. Real-time and sensitive detection of *Salmonella typhimurium* using an automated quartz crystal microbalance (QCM) instrument with nanoparticles amplification. Talanta 115, 761–767 (2013). 24054660 10.1016/j.talanta.2013.06.034

[b8] Fortes E. D., David J., Koeritzer B. & Wiedmann M. Validation of the 3M molecular detection system for the detection of *Listeria* in meat, seafood, dairy, and retail environments. J. Food Prot. 76, 874–878 (2013). 23643132 10.4315/0362-028X.JFP-12-552

[b9] Bunthof C. J., Bloemen K., Breeuwer P., Rombouts F. M. & Abee T. Flow cytometric assessment of viability of lactic acid bacteria. Appl. Environ. Microbiol. 67, 2326–2335 (2001). 11319119 10.1128/AEM.67.5.2326-2335.2001 PMC92874

[b10] Matsuoka H., Shigetomi T., Funabashi H., Saito M. & Igimi S. Tryptic soy medium is feasible for the in situ preparation of standards containing small defined numbers of microbial cells. J. Microbiol. Methods 93, 49–51 (2013). 23403310 10.1016/j.mimet.2013.01.021

[b11] Matsuoka H. *et al.* A flow cytometric method for the in situ preparation of standard materials of a small defined number of microbial cells with colony-forming potentiality. J. AOAC Int. 97, 479–483 (2014). 24830159 10.5740/jaoacint.13-302

[b12] Fujioka K., Geis P., Saito M. & Matsuoka H. Visualization of yeast single-cells on fabric surface with a fluorescent glucose and their isolation for culture. J. Ind. Microbiol. Biotechnol. 34, 685–688 (2007). 17566804 10.1007/s10295-007-0231-7

[b13] Matsuoka H. *et al.* Viable cell detection by the combined use of fluorescent glucose and fluorescent glycine. Biosci. Biotechnol. Biochem. 67, 2459–2462 (2003). 14646209 10.1271/bbb.67.2459

[b14] Wang X., Yamaguchi N., Someya T. & Nasu M. Rapid and automated enumeration of viable bacteria in compost using a micro-colony auto counting system. J. Microbiol. Methods 71, 1–6 (2007). 17669529 10.1016/j.mimet.2007.06.019

[b15] London R. *et al.* An automated system for rapid non-destructive enumeration of growing microbes. PLoS ONE 5, e8609 (2010). 20062794 10.1371/journal.pone.0008609 PMC2798718

[b16] Pandey R. *et al.* Live cell imaging of germination and outgrowth of individual *Bacillus subtilis* spores; the effect of heat stress quantitatively analyzed with SporeTtracker. PLoS ONE 8, e58972 (2013). 23536843 10.1371/journal.pone.0058972 PMC3607599

[b17] Levin-Reisman I. *et al.* Automated imaging with ScanLag reveals previously undetectable bacterial growth phenotypes. Nat. Methods 7, 737–739 (2010). 20676109 10.1038/nmeth.1485

[b18] Ogawa H. *et al.* Noise-free accurate count of microbial colonies by time-lapse shadow image analysis. J. Microbiol. Methods 91, 420–428 (2012). 23085533 10.1016/j.mimet.2012.09.028

[b19] ISO/IEC 17025. *General requirement for the competence of testing and calibration laboratories*. (ISO, 2005).

[b20] ISO 4832. Microbiology of food and animal feeding stuffs—Horizontal method for the enumeration of coliforms—Colony-count technique. (ISO, 2006).

[b21] Corry J. E. L., Jarvis B., Passmore S. & Hedges A. A. Critical review of measurement uncertainty in the enumeration of food microorganisms. Food Microbiol. 24, 230–253 (2007). 17188202 10.1016/j.fm.2006.05.003

[b22] Forster L. I. Conclusions on measurement uncertainty in microbiology. J. AOAC Int. 92, 312–319 (2009). 19382590

[b23] Nasu S, Inventor, Microbio Corporation, assignee. Photographing method and photographing device for subject in three-dimensional domain. Japanese patent. JP-4411109. 2009 Nov 20.

